# The Relationship between US Adults’ Misconceptions about COVID-19 Vaccines and Vaccination Preferences

**DOI:** 10.3390/vaccines9080901

**Published:** 2021-08-13

**Authors:** Sarah E. Kreps, Jillian L. Goldfarb, John S. Brownstein, Douglas L. Kriner

**Affiliations:** 1 Department of Government, Cornell University, Ithaca, NY 14850, USA; sarah.kreps@cornell.edu; 2 Department of Biological and Environmental Engineering, Cornell University, Ithaca, NY 14850, USA; jlg459@cornell.edu; 3 Computational Epidemiology Lab, Boston Children’s Hospital, Boston, MA 02115, USA; john.brownstein@childrens.harvard.edu

**Keywords:** COVID-19, public opinion, misconceptions, vaccine hesitancy

## Abstract

While mass vaccination has blunted the pandemic in the United States, pockets of vaccine hesitancy remain. Through a nationally representative survey of 1027 adult Americans conducted in February 2021, this study examined individual misconceptions about COVID-19 vaccine safety; the demographic factors associated with these misconceptions; and the relationship between misconceptions and willingness to vaccinate. Misconceptions about vaccine safety were widespread. A sizeable minority (40%) believed that vaccine side effects are commonly severe or somewhat severe; 85% significantly underestimated the size and scale of the clinical trials; and a sizeable share believed either that the vaccines contain live coronavirus (10%) or were unsure (38%), a proxy for fears that vaccination itself may cause infection. These misconceptions were particularly acute among Republicans, Blacks, individuals with lower levels of educational attainment, and unvaccinated individuals. Perceived side effect severity and underestimating the size of the clinical trials were both significantly associated with vaccine hesitancy.

## 1. Introduction

Although millions of Americans have been vaccinated against COVID-19, pockets of hesitancy remain [1]. As former Director of the Centers for Disease Control and Prevention Tom Frieden observed, “The biggest challenge to getting a COVID-19 vaccine into enough people’s arms won’t be scientific, technical or logistical; it will come from a lack of trust” [2]. Trust, he noted, would come down to whether the vaccine worked, was safe, and would be accessible to the public.

Merely being effective, safe, and accessible are necessary but not sufficient conditions for eliciting public trust, however. The public needs to believe that the vaccines meet those criteria [3]. Yet, misinformation about the virus has been prevalent since the beginning of the pandemic and risks hindering widespread vaccination [4,5]. Indeed, studies that exposed individuals to COVID-19 vaccine misinformation show a decline in self-reported willingness to vaccine [6].

Exposing individuals to misinformation could inadvertently persuade people to believe misinformation and dampen vaccine acceptance, raising ethical concerns [7]. We therefore take a different tack in studying how misconceptions about the vaccine influence the public’s vaccine preferences. We designed a study that identified individual misconceptions about vaccine safety; examined the demographic factors associated with these misconceptions; and modeled the relationship between the misconceptions and willingness to vaccinate. By analyzing the gaps in public understanding of the two COVID-19 vaccines that were authorized and in use in the United States in February 2021, Pfizer/BioNTech and Moderna, and whether those misconceptions are associated with individuals’ vaccination preferences, we identified opportunities for public outreach campaigns that may enhance vaccine uptake.

## 2. Materials and Methods

To evaluate attitudes about vaccine safety and the relationship between public misconceptions about COVID-19 vaccines and willingness to vaccinate, we conducted a survey online and via telephone from 11 to 15 February 2021 through the National Opinion Research Center (NORC), a non-partisan research institution at the University of Chicago. Our sample was recruited from the probability-based AmeriSpeak panel, which is representative of the US adult population [8]. NORC’s national sample frame provides coverage of approximately 97% of US households. Cornell University’s Institutional Review Board approved all protocols. 

Previous work has shown that perceptions of safety are the most important basis for COVID-19 vaccine acceptance and are a key factor explaining the racial and ethnic differences manifested on vaccination rates [9,10,11]. Our analysis measures the extent of misconceptions on three key dimensions regarding COVID-19 vaccine safety; explores how the extent of misperceptions varies across population subgroups; and tests whether these misconceptions are significant predictors of vaccine hesitancy, defined as delaying or refusing a vaccine despite its availability [12].

First, we asked respondents to estimate the size of the clinical trials as a proxy for safety concerns that have been expressed about the speed of COVID-19 development [13]. The question informed respondents that “Pfizer/BioNTech and Moderna, the companies that researched and produced the first two COVID-19 vaccines available to the public, conducted clinical trials to assess the safety of the vaccines.” It then asked: “If you had to guess, about how many individuals participated in these two studies combined?” We offered options of 1 to 1000; 1001 to 10,000; 10,001 to 25,000; 25,001 to 50,000; and the correct answer (at the time of the survey) of more than 50,000.

Second, we queried the perceived severity of vaccine side effects, also frequently expressed as a safety concern [14]. Respondents were told “it is typical for vaccines to have some side effects. Based on what you have heard about the COVID-19 vaccines, how severe do you think those potential side effects are?” Options included “severe”; “somewhat severe”; “not very severe”; “there are no side effects”. The CDC notes that “many people will have mild side effects after COVID-19 vaccination,” and that some people have no side effects at all [15] We also asked which side effects people “commonly experience after receiving a COVID-19 vaccine.”

Third, we probed another prominent misconception about safety: that the vaccine could actually transmit the virus, which the CDC includes at the top of their “myths and facts about COVID-19 vaccines.” To avoid exposing the respondent to any misinformation about the vaccine, we approached this question indirectly and stated that “some vaccines use a live, but weakened version of a virus to help people develop immunity. Other vaccines do not contain any live virus. To the best of your knowledge, do the COVID-19 vaccines currently available to the public contain any live coronavirus?” Response choices were: “yes, they contain live coronavirus”; “no, they do not contain live coronavirus”; and “don’t know/unsure”.

To measure vaccination hesitancy, we used a series of questions designed by the CDC. The first question asked respondents “have you ever received a COVID-19 vaccine.” Those who replied “yes” were then asked how many doses they had received. Those who replied that they had not yet received a COVID-19 vaccine were then asked “once a vaccine to prevent COVID-19 is available to you, would you…?” Answer choices were, “definitely get a vaccine,” “probably get a vaccine,” “probably not get a vaccine,” and “definitely not get a vaccine”. Respondents could also volunteer “don’t know” as a response. 

For each measure of each misconception, we calculated the percentage of our sample selecting each response option and 95% CIs, using survey sampling weights, via STATA 15. To examine the factors associated with these misconceptions, we estimated a pair of ordered logit regressions (for trial size estimates and perceived side effect severity) and a multinomial logit regression (for beliefs about whether the vaccine contains live coronavirus, with don’t know/unsure being the omitted baseline category). To assess associations between misconceptions about vaccine safety and willingness to vaccinate among the more than 80% of our sample that had not yet been vaccinated as of February 2021, we estimated an ordered logit regression including standard demographic controls [16].

## 3. Results

### 3.1. Prevalence of Safety Misconceptions

NORC invited 4526 panelists to take the survey, of whom 1027 accepted and completed the survey (completion rate: 22.7%). The demographic profile of our survey sample is presented in Table 1.

**Figure 1:**
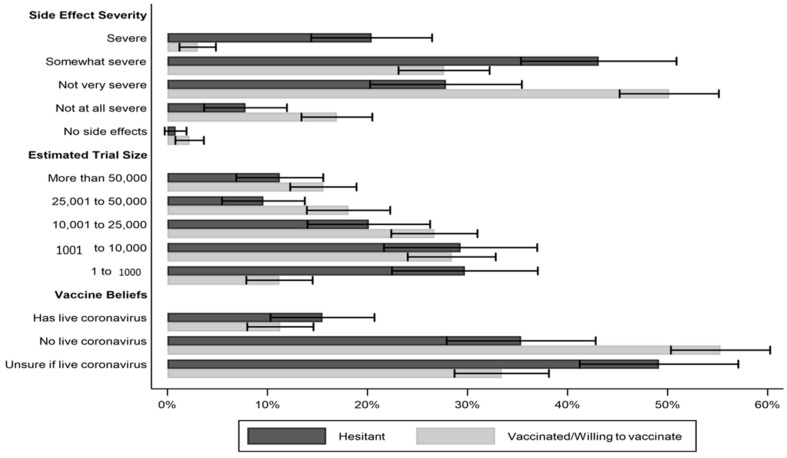
US adults’ perceptions of side effect severity, clinical trial size, and presence of live coronavirus in COVID-19 vaccines by vaccination status/hesitancy. Note: I-bars present 95% CIs.

A large percentage of our sample responded that COVID-19 side effects are either severe (7.9%, 95% CI: 5.8% to 10.1%) or somewhat severe (31.7%, 95% CI: 27.8% to 35.6%), indicating that respondents overestimated the severity of side effects compared to the CDC website, which (based on clinical evidence) informs people about “some minor side effects, which are normal signs that the vaccines are working.” As shown in Figure 1, there was a striking gap in perceived side effect severity between respondents who were already vaccinated or said they would “definitely” or “probably” be vaccinated when the opportunity arose and hesitant respondents—those who were not yet vaccinated and who said they would “definitely” or “probably” choose not to be vaccinated. Whereas 63.9% of hesitant respondents judged COVID-19 vaccine side effects to be severe or somewhat severe, just 30.4% of respondents who had already been vaccinated or who said they would be vaccinated when they had an opportunity to do so responded the same (*p* < 0.001, two-tailed test). By contrast, vaccinated and vaccine-accepting respondents were significantly more likely to say that side effects are not very severe than hesitant respondents (50.5% vs. 27.5%; *p* < 0.001, two-tailed test). 

Most Americans drastically underestimated the size of the Pfizer/BioNTech and Moderna clinical trials. Only 15.0% of respondents (95% CI: 12.2% to 17.7%) correctly responded that the two trials combined had enrolled more than 50,000 participants. The modal response, selected by 28.4% of respondents (95% CI: 24.6% to 32.2%), estimated that the trials enrolled only 1001 to 10,000 subjects. Here, again, we also observe a significant difference between vaccinated/willing to vaccinate respondents and hesitant respondents. Vaccinated/willing to vaccinate individuals were 13% more likely to select one of the two largest trial size estimates than were unvaccinated individuals (33.8% vs. 21.0%; difference in means is statistically significant, *p* < 0.01, two-tailed test). 

Many Americans were also uncertain as to whether the COVID-19 vaccines contain live coronavirus, a proxy for fears that recipients could actually contract the disease from the vaccine. Just under half of our sample (49.0%, 95% CI: 44.8% to 53.1%) correctly responded that the vaccines do not contain live coronavirus. However, 12.1% (95% CI: 9.4% to 14.8%) believed that the vaccines do contain live coronavirus, and the remainder were unsure. These fears were more common among hesitant individuals, though not significantly so. Whereas only 11.0% of vaccinated/willing to vaccinate individuals believed the vaccine contained live coronavirus, 15.3% of unvaccinated individuals expressed this belief (difference in means is not statistically significant, *p* = 0.17, two-tailed test). Vaccinated/willing to vaccinate individuals were significantly more likely to believe correctly that the vaccines do not contain live coronavirus than hesitant respondents (54.5% vs. 35.3% *p* < 0.001, two-tailed test).

### 3.2. Correlates of Misconceptions

To examine the correlates of holding each misconception about COVID-19 vaccines, we estimated a series of statistical models. Model 1 of Table 2 examines the factors associated with perceptions of side effect severity. Republicans were significantly more likely to perceive vaccine side effects as severe than were Democrats or independents (a Wald test confirms that the coefficient for the Republican indicator variable is statistically different from the coefficient for the Democratic indicator variable, *p* < 0.01, two-tailed test). Black respondents were also significantly more likely to perceive side effects as severe, all else being equal. 

As shown in Model 2, political partisanship and educational attainment were the most important predictors of beliefs about the size of the clinical trials. Republicans believed the trials were significantly smaller than did political independents or Democrats, all else being equal (a Wald test confirms that the coefficient for the Republican indicator variable is statistically different from the coefficient for the Democratic indicator variable, *p* < 0.01, two-tailed test). Respondents with greater levels of education were significantly more likely to believe the clinical trials involved many thousands of subjects than were respondents with less education. 

Model 3 presents results from a multinomial logit, which examines the factors associated with believing the vaccine either has or does not have live coronavirus; the unsure/don’t know category serves as the omitted baseline. Americans sixty or over were significantly less likely to believe the COVID-19 vaccines contain live coronavirus, all else being equal. Greater levels of educational attainment were positively associated with believing COVID-19 vaccines do not contain live coronavirus. Black and Latinx respondents were significantly less likely to believe that the vaccines do not contain live coronavirus. Even after controlling for a range of other factors, respondents who had already received at least one dose of the vaccine were significantly more likely to correctly believe the vaccines do not contain live coronavirus. 

### 3.3. Misconceptions and Vaccine Acceptance

The ordered logit regression analysis reported in Table 3 shows that misconceptions about side effect severity and estimates of clinical trial size were significantly associated with willingness to vaccinate. The more individuals were concerned about the severity of side effects, the less likely they were to report being likely to accept a COVID-19 vaccine. The higher a respondent’s estimate of the number of individuals in the clinical trials, the more likely he or she was to report willingness to vaccinate. Believing that the vaccine contains live coronavirus was not significantly associated with vaccination intention. Associations for control variables largely correspond to those observed in other studies of COVID-19 vaccines [17].

## 4. Conclusions

Vaccine willingness is a calculation of the perceived risk posed by the virus and the confidence and convenience of the vaccine [18]. Higher perceived side effects increase the perceived risk of the vaccine, which could in turn affect the payoff of vaccinating and lower the likelihood of vaccinating. We found that many Americans perceive vaccine side effects to be more severe than the empirical evidence from clinical trials suggests. We saw strong evidence of a cost–benefit logic with a strong negative association between vaccine acceptance and perceived side effect severity. Further, we found that Republicans and Blacks, who have expressed vaccine hesitancy or lagged in vaccination rates [9,19,20,21], are more likely to hold the misconception that COVID-19 vaccine side effects are severe. 

Individuals may indeed differ in what they consider to be “severe” compared to the CDC, but perception appears to have a meaningful impact on vaccine preferences. Open-ended responses to our question about the side effects people “commonly experience” with the COVID-19 vaccine shed additional light on how people thought about “severity” and its effect on vaccination behavior. Taking the most extreme side effect that respondents identified, death, we found that 37 individuals (close to 4% of the sample) cited death as a common side effect; of these, 31 said they would probably or definitely not be vaccinated. Given the statistical rarity of death associated with COVID-19 vaccination, especially relative to the mortality rate among those who contract COVID-19, the strong relationship between the perceived severity of side effects and vaccine preferences points to a need to reinforce educational efforts informing the public that serious side effects such as fatalities are exceedingly rare.

In addition to misconceptions about side effects, we found that a significant share of Americans systematically under-estimate the size of the COVID-19 vaccine clinical trials. Previous studies showed that Emergency Use Authorization (EUA) dampens vaccine willingness [16,22], and that the size of this effect may have increased over time [23]. Our findings suggest that perceptions about a perfunctory vaccine development continue to weigh negatively on vaccine willingness. Education efforts that emphasize the size of the trials may offset concerns (among those who are less educated in general and less knowledgeable on the specifics of the clinical trials) that the clinical trials were too small to uncover potential serious consequences of vaccination. 

An important limitation of our study is that we conducted the survey in February 2021, before the significant surge in vaccination that began in mid-February 2021. As more people within one’s social network become vaccinated and more empirical evidence about vaccine safety accrues, these misconceptions might have receded. On the other hand, the Biden Administration observed in July 2021 that social media is “killing people” because of the endemic COVID-19 misinformation that persists, renewing questions about the relationship between vaccine misconceptions and vaccine behavior [24]. Future research should examine the persistence of vaccine misconceptions and their relationship to vaccine acceptance as the vaccination campaign progresses to better understand persistent pockets of hesitancy and inform more targeted, effective public health outreach.

## Figures and Tables

**Table 1:** Survey sample demographics.

	N	Percentage
Age		
18–29	162	(16%)
30–44	315	(31%)
45–59	238	(23%)
>=60	312	(30%)
**Gender**		
Male	504	(49%)
Female	523	(51%)
**Race/Ethnicity**		
White	669	(65%)
Black	114	(11%)
Latino	153	(15%)
Asian	33	(4%)
Other	58	(6%)
**Education**		
Less than High School	44	(4%)
High School/GED	156	(15%)
Some College	404	(39%)
4-Year College Degree	233	(23%)
Graduate School	190	(19%)
**Income**		
<$30,000	242	(24%)
$30,000 to $59,999	297	(29%)
$60,000 to $99,999	261	(25%)
>=$100,000	227	(22%)
**Political Partisanship**		
Democrat (includes leaners)	495	(48%)
Republican (includes leaners)	403	(39%)
Independent	129	(13%)
**Vaccination Status**		
Vaccinated (at least one dose)	196	(19%)
Not vaccinated	830	(81%)
**Vaccination Intention (Unvaccinated Only)**		
Definitely get a vaccine	358	(43%)
Probably get a vaccine	184	(22%)
Probably not get a vaccine	156	(19%)
Definitely not get a vaccine	129	(16%)

Note: Percentages may not sum to 100% because of rounding.

**Table 2:** Factors associated with clinical trial size estimates, beliefs vaccine contains live coronavirus, and perceived side effect severity.

	(1)			(2)			(3)					
	Side Effect Severity			Trial Size Estimate			Has Live Virus			No Live Virus		
	Coef.	*p*-Value	95% CI	Coef.	*p*-Value	95% CI	Coef.	*p*-Value	95% CI	Coef.	*p*-Value	95% CI
Democrat	0.04	(0.88)	(−0.43–0.50)	−0.07	(0.76)	(−0.51–0.38)	0.15	(0.73)	(−0.72–1.02)	−0.04	(0.90)	(−0.71–0.63)
Republican	0.53 *	(0.03)	(0.04–1.02)	−0.63 **	(0.01)	(−1.11–−0.15)	−0.34	(0.43)	(−1.21–0.52)	−0.37	(0.28)	(−1.04–0.30)
Female	0.20	(0.19)	(−0.10–0.51)	−0.12	(0.43)	(−0.42–0.18)	−0.08	(0.79)	(−0.64–0.48)	0.02	(0.92)	(−0.37–0.41)
Age: 30–44	0.26	(0.27)	(−0.20–0.72)	−0.04	(0.86)	(−0.48–0.40)	−0.06	(0.88)	(−0.82–0.70)	−0.14	(0.63)	(−0.73–0.44)
Age: 45–59	0.10	(0.71)	(−0.43–0.63)	−0.22	(0.36)	(−0.67–0.24)	−0.01	(0.98)	(−0.86–0.84)	−0.03	(0.92)	(−0.66–0.60)
Age: 60+	−0.17	(0.47)	(−0.63–0.29)	0.07	(0.77)	(−0.39–0.52)	−1.07 *	(0.02)	(−1.94–−0.20)	−0.33	(0.28)	(−0.92–0.27)
Education	−0.13	(0.07)	(−0.26–0.01)	0.24 **	(0.00)	(0.08–0.39)	0.19	(0.13)	(−0.06–0.45)	0.45 **	(0.00)	(0.27–0.63)
Black	0.71 *	(0.02)	(0.14–1.28)	−0.40	(0.18)	(−1.00–0.19)	0.12	(0.74)	(−0.60–0.85)	−1.57 **	(0.00)	(−2.22–−0.91)
Latinx	0.16	(0.53)	(−0.33–0.64)	−0.35	(0.10)	(−0.77–0.07)	0.17	(0.65)	(−0.57–0.90)	−0.62 *	(0.03)	(−1.17–−0.07)
Vaccinated	−0.35	(0.06)	(−0.72–0.01)	0.20	(0.32)	(−0.19–0.58)	−0.50	(0.25)	(−1.35–0.35)	0.90 **	(0.00)	(0.40–1.40)
Constant							−1.35 *	(0.03)	(−2.58–−0.13)	−0.69	(0.19)	−1.73–0.34)
Observations	1010			995			1022			1022		

Note: Models 1 and 2 are ordered logit regressions using 5-point dependent variables. Model 3 is a multinomial logit and the “unsure/don’t know” response serves as the omitted baseline category. Table reports regression coefficients with standard errors in parentheses. All significance tests are two-tailed. ** *p* < 0.01, * *p* < 0.05.

**Table 3:** Associations between misconceptions, demographics, and willingness to vaccinate.

	Coefficient	*p*-Value	95% CI
Estimated size of clinical trials	0.16 *	(0.03)	(0.02–0.30)
Expected side effect severity	−1.05 **	(0.00)	(−1.29–−0.80)
Vax has live coronavirus	0.35	(0.24)	(−0.24–0.94)
Democrat	0.43	(0.15)	(−0.15–1.01)
Republican	−0.73 *	(0.01)	(−1.30–0.16)
Female	−0.31	(0.09)	(−0.67–0.05)
Age: 30–44	0.26	(0.33)	(−0.26–0.78)
Age: 45–59	0.49	(0.10)	(−0.09–1.07)
Age: 60+	0.89 **	(0.00)	(0.31–1.46)
Education	0.21 *	(0.01)	(0.05–0.36)
Black	−1.17 **	(0.00)	(−1.73–0.61)
Latinx	−0.38	(0.17)	(−0.93–0.17)
Observations	796		

Note: Model is an ordered logit regression using a 4-point dependent variable (definitely will vaccinate to definitely will not vaccinate). Table reports regression coefficients, *p*-values, and 95% confidence intervals. All significance tests are two-tailed. ** *p* < 0.01, * *p* < 0.05.

## Data Availability

All replication data and code is available at the Harvard Dataverse: https://doi.org/10.7910/DVN/FJL4QC (accessed on 13 August 2021).

## Highlights

Misconceptions about COVID-19 vaccine safety are common among U.S. public; misconceptions are strongest among hesitant groups, including racial minorities and Republicans; beliefs that clinical trials were small and side effects are severe are significantly associated with vaccine hesitancy.
